# Eradication of Peste des Petits Ruminants Virus and the Wildlife-Livestock Interface

**DOI:** 10.3389/fvets.2020.00050

**Published:** 2020-03-13

**Authors:** Amanda E. Fine, Mathieu Pruvot, Camilla T. O. Benfield, Alexandre Caron, Giovanni Cattoli, Philippe Chardonnet, Maurizio Dioli, Thomas Dulu, Martin Gilbert, Richard Kock, Juan Lubroth, Jeffrey C. Mariner, Stephane Ostrowski, Satya Parida, Sasan Fereidouni, Enkhtuvshin Shiilegdamba, Jonathan M. Sleeman, Claudia Schulz, Jean-Jacques Soula, Yves Van der Stede, Berhe G. Tekola, Chris Walzer, Steffen Zuther, Felix Njeumi, Meeting Participants

**Affiliations:** ^1^Wildlife Conservation Society, Health Program, Bronx, NY, United States; ^2^Royal Veterinary College, University of London, London, United Kingdom; ^3^ASTRE, University of Montpellier, CIRAD, INRA, Montpellier, France; ^4^Veterinary Faculty, Eduardo Mondlane University, Maputo, Mozambique; ^5^Animal Production and Health Laboratory, Joint FAO/IAEA Division for Nuclear Applications in Food and Agriculture, International Atomic Energy Agency, Seibersdorf, Austria; ^6^Antelope Specialist Group, International Union for Conservation of Nature, Species Survival Commission, Gland, Switzerland; ^7^Independent Camel Specialist, Khartoum, Sudan; ^8^State Department of Livestock, Ministry of Agriculture, Livestock and Fisheries, Nairobi, Kenya; ^9^Department of Population Medicine and Diagnostic Services, College of Veterinary Medicine, Cornell University, Ithaca, NY, United States; ^10^Animal Health Service, Animal Production and Health Division, Food and Agriculture Organization of the United Nations, Rome, Italy; ^11^Cummings School of Veterinary Medicine, Tufts University, Grafton, MA, United States; ^12^Vaccine Differentiation Department, Pirbright Institute, Woking, United Kingdom; ^13^Department of Interdisciplinary Life Sciences, Research Institute of Wildlife Ecology, University of Veterinary Medicine, Vienna, Austria; ^14^Wildlife Conservation Society, Mongolia Country Program, Ulaanbaatar, Mongolia; ^15^US Geological Survey, National Wildlife Health Center, Madison, WI, United States; ^16^Working Group on Wildlife, Office International des Epizooties/World Organisation for Animal Health, Paris, France; ^17^Research Center for Emerging Infections and Zoonoses, University of Veterinary Medicine Hannover, Hanover, Germany; ^18^FAO-OIE GEP PPR Secretariat, Food and Agriculture Organization of the United Nations, Rome, Italy; ^19^European Food Safety Authority, Parma, Italy; ^20^Office of the Director, Animal Production and Health Division, Food and Agriculture Organization of the United Nations, Rome, Italy; ^21^Association for the Conservation of Biodiversity of Kazakhstan, Nur-Sultan, Kazakhstan; ^22^Frankfurt Zoological Society, Frankfurt, Germany

**Keywords:** wildlife-livestock interface, peste des petits ruminants, small ruminant morbillivirus, global eradication, integrated management, wildlife conservation, one health

## Abstract

Growing evidence suggests that multiple wildlife species can be infected with peste des petits ruminants virus (PPRV), with important consequences for the potential maintenance of PPRV in communities of susceptible hosts, and the threat that PPRV may pose to the conservation of wildlife populations and resilience of ecosystems. Significant knowledge gaps in the epidemiology of PPRV across the ruminant community (wildlife and domestic), and the understanding of infection in wildlife and other atypical host species groups (e.g., camelidae, suidae, and bovinae) hinder our ability to apply necessary integrated disease control and management interventions at the wildlife-livestock interface. Similarly, knowledge gaps limit the inclusion of wildlife in the FAO/OIE Global Strategy for the Control and Eradication of PPR, and the framework of activities in the PPR Global Eradication Programme that lays the foundation for eradicating PPR through national and regional efforts. This article reports on the first international meeting on, “Controlling PPR at the livestock-wildlife interface,” held in Rome, Italy, March 27–29, 2019. A large group representing national and international institutions discussed recent advances in our understanding of PPRV in wildlife, identified knowledge gaps and research priorities, and formulated recommendations. The need for a better understanding of PPRV epidemiology at the wildlife-livestock interface to support the integration of wildlife into PPR eradication efforts was highlighted by meeting participants along with the reminder that PPR eradication and wildlife conservation need not be viewed as competing priorities, but instead constitute two requisites of healthy socio-ecological systems.

## Introduction

Peste des petits ruminants (PPR) is a widespread and devastating disease of domestic and wild artiodactyls caused by peste des petits ruminants virus (PPRV; small ruminant morbillivirus) (1). Among domestic animals, goats and sheep are primarily affected, representing a threat to the primary source of livelihoods for 300 million rural families globally (2), and an estimated US$2.1 billion in economic losses per year (3). As a response, the Food and Agriculture Organization of the United Nations (FAO) and the World Organisation for Animal Health (OIE) endorsed the Global Strategy for the Control and Eradication of PPR (PPR GCES), and launched the PPR Global Eradication Programme (PPR GEP), to eradicate PPRV by 2030 (2, 3). To date, PPR GEP has focused on the surveillance and control of PPR in affected livestock. Although a range of wildlife hosts are known to be susceptible to PPRV (4, 5), the role of wildlife has been assumed, as was the case with rinderpest, to play a minor epidemiological role (6, 7). As a result, PPRV ecology, dynamics, and impact across susceptible artiodactyl communities have not been sufficiently considered (8).

PPRV outbreaks in free-ranging wild artiodactyls can result in severe mortality and threaten wildlife populations and ecosystem stability (9–12), although the full impact on biodiversity conservation remains to be determined. In endemic situations, such as in East Africa, serological responses to PPRV in wildlife indicate widespread spillover at the wildlife-livestock interface, but no overt disease (13). In Asia, PPR outbreaks have impacted wildlife populations, as documented in Mongolia in 2017 with large-scale mortality in the critically endangered saiga antelope (*Saiga tatarica mongolica*) (14). The potential role of wildlife species as maintenance hosts for PPRV in these different ecosystems is unknown. It is also unclear what factors are driving the apparent difference in disease expression between Asian and African wildlife. The expansion of PPR into free-ranging wildlife, continental Asia, and eastern Europe are major concerns that negatively impact biodiversity, dim the vision of a PPR-free world by 2030 (15), and threaten the realization of UN Sustainable Development Goals (SDGs 1, 2, 3, 5, and 15).

In recognition of the threat to PPR eradication, and to galvanize broader support for investigation and action at the wildlife-livestock interface, a meeting, “Controlling PPR at the livestock-wildlife interface,” was convened March 27–29, 2019, in Rome, Italy. The meeting was co-organized by FAO, OIE, Wildlife Conservation Society, and Royal Veterinary College, with coordination and support provided by Science for Nature and People Partnership (SNAPP) and the FAO/OIE PPR GEP Secretariat. Invited experts, representing diverse national governments and international institutions, focused on: (1) discussing recent scientific knowledge on PPR at the wildlife-livestock interface, (2) identifying significant knowledge gaps and research priorities on PPRV and wildlife, and (3) drawing lessons learned from PPR control across the ruminant community (wildlife and domestic animals). This article condenses the meeting report and highlights key research and policy priorities, as well as recommendations.

## Recent Scientific Insights on PPR at the Wildlife-Livestock Interface

**Figure 1:**
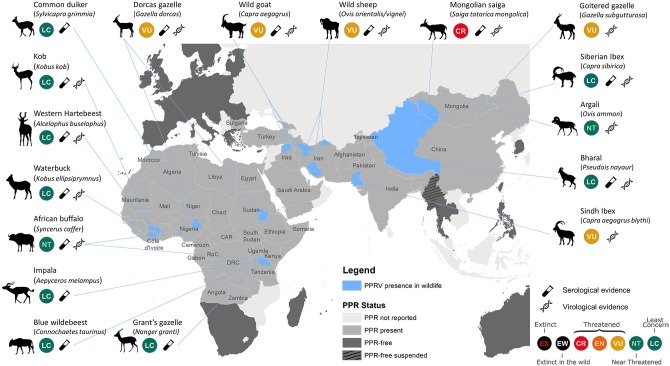
Map illustrating published reports of PPRV detection in free-ranging wildlife species. Data compiled from published reviews and reports (excluding insufficiently documented cases) (4, 5, 10–14, 16–22), official PPR status from World Organization for Animal Health (OIE, www.oie.int/en/animal-health-in-the-world/ppr-portal), and International Union for Conservation of Nature (IUCN) Red List species status (www.iucnredlist.org); CR, Critically Endangered; EN, Endangered; VU, Vulnerable. Areas of PPR detection in wildlife (in blue) are restricted to the states/provinces where wildlife cases were detected. The level of evidence is provided to distinguish serological evidence from virological (i.e., virus isolation, Ag-ELISA, and/or RT-PCR) evidence. PPRV was detected in Argali sheep, Goitered gazelle, and Siberian ibex in both China and Mongolia.

Recent reviews and case reports have established PPR as a disease of both domestic small ruminants and wild artiodactyls (4, 5, 10–14, 16–23) (Figure 1). PPRV continues to expand geographically in unvaccinated susceptible populations of domestic small ruminants, facilitating spillover of virus where domestic and wild artiodactyl species coexist and share resources. PPR caused high morbidity and mortality in the Mongolian saiga antelope, contributing to an 80% reduction of the population, and threatening this subspecies with extinction (9, 14). Clinical PPRV infection has been documented in other threatened wild artiodactyls in Asia. In Pakistan, cases were identified in Sindh ibex (*Capra aegagrus blythi*) (10), and seroconversion was detected in free-ranging domestic yaks (*Bos grunniens*) (24). Recent outbreaks in China involved ibex (*Capra ibex sibirica*), argali sheep (*Ovis ammon*), and goitered gazelle (*Gazella subgutturosa*) (11, 18). Wild goats (*C. aegagrus*) were affected in Iraqi Kurdistan (20), and both wild goats and wild sheep (*O. orientalis/vignei*) were repeatedly impacted in Iran following outbreaks in livestock (12).

In a number of PPRV endemic countries in Africa, there is growing serological evidence of repeated PPRV infection of diverse wildlife species (25), but no overt disease confirmed in free-ranging populations. In Tanzania, sero-positivity was confirmed in African buffalo (*Syncerus caffer caffer*), blue wildebeest (*Connochaetes taurinus*), impala (*Aepyceros melampus*), common tsessebe (*Damaliscus lunatus*), and Grant's gazelle (*Nanger granti*), but with little understanding of the role of these species in PPR epidemiology (13, 26). Other anecdotal reports include seroconversion to PPRV in the West African giraffe (*Giraffa camelopardalis* ssp. *peralta*) in Niger (Chardonnet P., personal communication), and viral detection in dorcas gazelles (*Gazella dorcas*) in Sudan (16). Recent research into atypical hosts for PPRV suggest that domestic pigs and wild boar (*Sus scrofa*), and possibly warthogs *(Phacochoerus africanus*), are competent hosts for the virus, with sufficient viral replication and shedding to enable PPRV transmission. Their role in natural systems needs further consideration (27). Multiple reports suggest that dromedaries (*Camelus dromedarius*) are susceptible to PPRV infection and express disease clinically, as observed in Iran, Ethiopia, and Sudan (28–30), though recent PPRV experimental infection trials with camelids resulted in no clinical disease or shedding of PPRV (31). Meeting participants discussed the potential of roe deer (*Capreolus capreolus*) and wild boar facilitating the introduction and spread of PPRV into the European Union, though there is no evidence to suggest that this has occurred.

## Research Gaps and Priorities

Research gaps and priorities identified grouped into four themes: (1) Diagnostic tools; (2) Risk of PPRV infection in diverse wildlife populations; (3) Epidemiological role of wildlife and impact on wildlife conservation; and (4) Ecological perspectives on PPR at wildlife-livestock interfaces in complex socio-ecological systems.

### PPRV Diagnostics in Wildlife

Diagnostic tools for PPRV detection, primarily developed for livestock species, have not been standardized and adequately validated for wildlife. This results in uncertainty regarding the validity of individual-level diagnostics, and most importantly, of population level inference (32). For serological diagnostic tools, a trade-off was highlighted between practicality in most laboratory settings and validation for wildlife species. All available diagnostic options [Virus Neutralization Test, blocking ELISA, pseudotype-based neutralization assays, and PPR-Luciferase Immunoprecipitation System (33, 34)] have value and shortcomings that must be recognized. Moving forward, clear guidelines and standards for application and interpretation of PPR diagnostic tests in wildlife species need to be established. Parallel and replicated testing of samples with multiple diagnostic methods will contribute to our understanding of the respective performance and accuracy of each test (32, 35).

Diagnostic tools to detect viral shedding (e.g., antigen ELISA, qRT-PCR), may facilitate the identification of populations of greatest importance to PPR eradication. Molecular epidemiology using genomic data has the potential to clarify the roles of wildlife in PPRV circulation, direction of transmission at wildlife-livestock interfaces (36), and how viral evolution may alter host range and virulence (37). Thus, high resolution genetic data (i.e., full PPRV gene or genome) from a range of domestic and wild species is needed. In many countries, access to the required sequencing technology is limited, compounded by the difficulty in transporting wildlife samples across international borders due to Convention on International Trade in Endangered Species of Wild Fauna and Flora, and Nagoya Protocol regulations. Challenges also include practical and ethical requirements associated with obtaining samples from wildlife.

### Identifying Risk of PPR in Wildlife Populations

Recognizing that many wildlife species are susceptible to PPRV infection, there are distinct and potentially conflicting criteria for identifying populations requiring additional attention. Wildlife populations of greatest importance to eradication efforts are those populations/communities that contribute to PPRV maintenance and transmission, alone or in interaction with domestic populations. Non-maintenance wildlife populations may be sympatric with, and potentially transmit PPRV to, other wild species with wider ranges and capacity for virus transmission to livestock, thereby acting as bridge hosts (38). Wildlife populations of greatest conservation concern may or may not play an important role in PPR maintenance. However, the impact of PPR in these endangered populations may be devastating, as illustrated by the outbreak in Mongolian saiga (14). Many wild mountain caprine species exist in small fragmented populations, making them highly vulnerable to extirpation as a result of disease outbreaks (9).

Therefore, it is important to consider the entire host community and employ transparent prioritization criteria when allocating resources for PPR research and eradication. Diverse risk assessment approaches, including spatio-temporal risk mapping, can guide prioritization at wildlife-livestock interfaces (39, 40). Participatory epidemiology supports this process, facilitates community engagement, and generates broader support for management decisions (41–43).

### The Epidemiological Role of Wildlife and PPR Impact on Wildlife

Information on PPR in wildlife has mainly focused on reporting occurrence in new species, with little data on the virus ecology in these systems (8), the significance for disease control, or threat to wildlife conservation. The small number of samples collected for laboratory analysis during disease outbreaks in wildlife is a further constraint. A greater understanding of the epidemiology of PPRV at the wildlife-livestock interface is required to formulate science-based management options that support eradication efforts and protect biodiversity. Knowledge gaps exist across all steps of the spillover process: susceptibility of wildlife hosts, transmission mechanisms, and the ability of new host species to maintain infection (44).

Assessing the impact of PPRV on the conservation of wildlife populations requires urgent attention. Initial reports of disease impacts on wildlife are often based on direct counts of dead animals, leading to underestimates of impact at the population level (14, 32). Species-specific wildlife survey methods, that account for the probability of detection, must be adopted and applied consistently to support the accurate documentation of PPRV impact on wildlife populations. Integration of this information using dynamic models of within- and between-host transmission will clarify the role of wildlife in PPRV epidemiology, the impact of wildlife hosts on eradication strategies, and the expected short- and long-term impact of PPRV on wild ungulate communities.

The lack of species-specific information on susceptibility, amount and duration of viral excretion, and dynamics of immune response, hinders interpretation of the epidemiological role of diverse artiodactyl species. Experimental infection studies illustrate the value of *ex situ* research in this area (27), acknowledging the expense and ethical considerations of conducting this work. Serological monitoring of vaccinated captive wildlife and atypical host species would provide information about the immunogenicity of available vaccines, and the dynamics of the immune response, which can be used to improve inference from serological data obtained via routine monitoring (45).

There are critical gaps in our understanding of mechanisms of transmission between livestock and wildlife, including the potential for indirect transmission (e.g., via fomites, pasture, feed, water, and mechanical insect vectors). This requires research on viral viability on/in various substrates (e.g., water, soil, mineral licks, hair coat, feces, and carcasses) and under a range of environmental conditions (e.g., temperature, humidity, ultraviolet exposure, water turbidity, and salinity). Detailed descriptions of wildlife-livestock interactions using spatio-temporal analysis can further assess the contribution of these transmission routes to inter-species transmission (46–48), thereby identifying potential prevention and control measures (44).

Most importantly, the participants stressed the need to learn from PPR interventions by including the simultaneous monitoring of wildlife species in pre- and post-vaccination monitoring. Vaccination of livestock in critical ecosystems will create opportunities to answer important questions about the potential of in-contact wildlife/atypical host populations to maintain virus, or the potential for enhanced livestock vaccination to prevent spillover into wild artiodactyls. These opportunities for quasi-experiments have been recognized as crucial for identifying reservoirs of infection in other multi-host systems (49) and need to be identified in advance to benefit our understanding of the dynamics of PPRV between livestock and wildlife/atypical hosts.

### Broader Ecosystem-Level Perspective

The meeting participants highlighted the need to look beyond the multi-host epidemiological systems to include a broader examination of the socio-ecological determinants of PPRV dynamics, and ecosystem-level impacts. As PPRV (or other pathogens) drive wildlife populations to local extinction, bottom-up (e.g., on predators) and top-down (e.g., on plant communities) effects must be expected (50), which may considerably alter grazing ecosystems. In systems where PPRV was observed to spillover into wildlife, anthropogenic factors should be considered, including the effects of competition for resources between domestic and wild ungulates due to increasing livestock numbers, or of different livestock management systems. The occurrence, spread, and expression of PPRV may be driven by other environmental, climatic, economic, and social factors, which may not be adequately addressed by conventional disease control approaches (44).

Other outstanding questions deserve attention: Is eradication achievable without explicitly including wildlife in control strategies? While the eradication of PPRV in livestock is a desirable outcome, will eradication have a net positive or negative impact for sympatric wild ungulates? May this net impact vary through the different stages of the eradication process? Are there strategies that can optimize control effectiveness, wildlife protection, and long-term socio-economic outcomes? All these questions require multi-disciplinary, trans-sectoral, and collaborative approaches, combining amongst others, veterinary science, epidemiology, ecology, and social sciences with strong community engagement via participatory approaches.

## Lessons Learned and Recommendations

**Figure 2:**
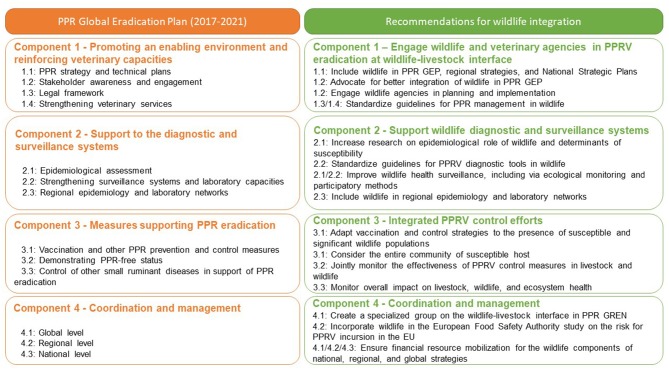
Main components of the PPR Global Eradication Programme (PPR GEP) **(left column)** and suggested additions of wildlife specific activities to the four main components **(right column)**.

The PPR GEP is a framework with planned activities over an initial 5-year phase (2017–2021) covering four major components designed to lay the foundation for eradicating PPRV (2). Answers to the key research questions outlined above will be required to develop effective PPR surveillance and diagnostic systems (Component 2 of PPR GEP) and to design effective measures supporting PPR eradication (Component 3 of PPR GEP) at the wildlife-livestock interface. Meeting participants observed that the current PPR GEP does not include wildlife species or considerations of impacts on biodiversity (UN SDG 15—Life on Land). Moreover, there is limited information available and a lack of guidelines for policy makers and practitioners on the investigation and control of PPR in wildlife. Consequently, the meeting participants formulated the following recommendations to be addressed now and considered for inclusion in successive phases of PPR GEP (Figure 2).

### Recommendations for Component 1: Promoting an Enabling Environment and Reinforcing Veterinary Capacities

- Provide policy makers and practitioners with internationally recognized and standardized guidelines for addressing PPR in wildlife. Meeting participants recommended that the Working Group on Wildlife of the OIE and the PPR Global Research and Expertise Network (PPR GREN) draft joint FAO/OIE guidelines for the surveillance, control, and prevention of PPR in wildlife populations^1^.
- Integrate wildlife into PPR GCES. The next PPR GEP (2022–2027) document should incorporate wildlife across the four main components of PPR GEP.
- Include wildlife populations in planning of PPR surveillance, control, and eradication activities in National Strategic Plans (NSP) and regional strategies.
- Engage wildlife practitioners (including OIE National Focal Point on Wildlife) and agencies with responsibility for protecting wildlife in PPR GEP training and capacity building initiatives, including the PPR monitoring and assessment tool (PMAT).
- Continue advocating for integration of wildlife into the PPR GCES, including by groups such as the IUCN Species Survival Commission Wildlife Health Specialist Group, to protect biodiversity and the goal of PPR eradication by 2030.

### Recommendations for Component 2: Support for Surveillance and Diagnostic Systems

- Establish and share clear guidelines and standards for application of PPR diagnostics tests in wildlife species via OIE.
- Improve wildlife health surveillance systems and systematically conduct thorough wildlife disease outbreak investigations, in particular at the wildlife-livestock interface. Standard ecological monitoring methods, including species-specific wildlife survey protocols, and participatory disease surveillance methods should be expanded to improve our understanding of PPRV at wildlife-livestock interfaces and optimize management strategies.

### Recommendations for Component 3: Measures Supporting PPR Eradication

- Identify wild host populations at risk of PPR infection and coordinate between national veterinary and environmental authorities to prioritize targeted vaccination at these wildlife-livestock interfaces.
- Plan and implement vaccination campaigns in concert with communities of livestock owners informed by an understanding of PPRV epidemiology across the ruminant community.
- Assess the immunogenicity/efficacy of PPRV vaccination in susceptible species other than domestic small ruminants.
- Identify science-based alternative management strategies to prevent disease spillover, while avoiding negative impacts on wildlife populations.
- Assess the impact of PPRV control measures by monitoring both livestock and wildlife populations.

### Recommendations for Component 4: Coordination and Management

- Establish a specialized group of the PPR GREN on wildlife to promote and support on-going research on PPR at the wildlife-livestock interface^2^.
- Support the incorporation of knowledge on PPR at the wildlife-livestock interface in European Food Safety Authority (EFSA) risk assessments for PPRV incursion in the EU.
- Advocate with donors and partners to ensure adequate financial resource mobilization for implementation of PPR GEP (including wildlife components) at national, regional, and global levels.

## Conclusion

Recent reports and research at the wildlife-livestock interface make a strong case that wildlife hosts can no longer be ignored in the epidemiology of PPRV. Evidence of transmission between wildlife and livestock may delay PPRV eradication goals. PPRV is also a clear conservation threat to diverse, ecologically important, and often threatened wild species. Strikingly, scientific evidence to formally assess these impacts is lacking across all ecosystems where domestic and wild susceptible hosts coexist. This knowledge gap correlates with a policy gap, as wildlife has until now largely been absent from the PPR GEP framework and National Strategic Plans. We believe that both gaps need to be addressed in order to meet global PPRV eradication goals while protecting global biodiversity. We acknowledge the challenge of resource allocation, but highlight that PPRV eradication and wildlife conservation need not be viewed as competing priorities, but are instead two requisites of healthy socio-ecological systems. This will not only require a better understanding of these systems, but also the long-term commitment, dialogue, and collaboration of diverse stakeholders toward these goals.

## Data Availability Statement

The datasets generated for this study are available on request to the corresponding author.

## Author Contributions

AF, CB, AC, GC, PC, MD, TD, MG, RK, JL, JM, SO, SP, SF, ES, JS, CS, J-JS, YV, BT, CW, SZ, and FN contributed significant content to the PPR wildlife-livestock interface components of the meeting. Authors listed as Meeting Participants participated in the meeting and group discussions. AF, RK, CW, FN, and J-JS prepared the initial meeting report. MP wrote the first draft of the manuscript adapted from the meeting report and prepared figures. AF and MP completed the manuscript. All authors contributed to manuscript revision, read and approved the submitted version.

## Meeting Participants

Ibrahim Gashash AHMED (AU-IBAR, Kenya), M. AFZAL (FAO Pakistan, Pakistan), Elmira AKMATOVA (Kyrgyz Research Institute of Veterinary, Kyrgyzstan), Shereefa AL-SALEM (Biodiversity Conservation Department, Environment Public Authority, Kuwait), Chrisostom AYEBAZIBWE (FAO Uganda, Uganda), Klaas DIETZE (Friedrich-Loeffler-Institut, Federal Research Institute for Animal Health, Germany), Emma GORENBERG (US Fish and Wildlife Service, USA), Jörg HARTUNG (Germany), Akiko KAMATA (FAO Rome, Italy), Julius KEYYU (Tanzania), R. Scott LARSEN (Denver Zoological Foundation, USA), Junaidu A. MAINA (J M Global Associates Ltd, Nigeria), Thomas C. METTENLEITER (Friedrich-Loeffler-Institut, Germany), Alexandra MITEVA (Bulgarian Food Safety Agency, Bulgaria), David MODRY (University of Veterinary and Pharmaceutical Sciences Brno, Czech Republic), Rajabali MUZAFFAR (Veterinary Institute of Tajik Academy of Agricultural Sciences, Tajikistan), Mathew MUTINDA (Veterinary Department of the Kenya Wildlife Service, Kenya), Sophycate NJUE (FAO Kenya, Kenya), Hervé PETIT (Agronomes et Vétérinaires Sans Frontières, France), Batsaikhan SODNOM (Mongolian Ministry of Food, Agriculture & Light Industry, Mongolia), Gijs VANTKLOOSTER (FAO Ethiopia, Ethiopia), Ganchimeg WINGARD (Denver Zoological Foundation, USA), Peregrine WOLFF (Nevada Department of Wildlife/Wild Sheep Foundation, USA).

### Conflict of Interest

The authors declare that this research was conducted in the absence of any commercial or financial relationships that could be construed as a potential conflict of interest.

## Footnotes

^1^At the time of writing this manuscript, guidelines are in preparation with sections on programme planning and governance, surveillance and outbreak investigation, standardization and data management, laboratory diagnostics, risk assessments, control and prevention options, and risk communication.

^2^The wildlife group was formally created at the PPR GREN meeting in Nairobi (13th-15th November 2019). The GREN wildlife group validated the research priorities outlined in this manuscript, stating that adopting a holistic systems approach in PPRV eradication will optimize outcomes for human communities, their livestock, and biodiversity.
